# Transitional Life Events in Friedreich Ataxia: Differential Age at Onset Perspectives

**DOI:** 10.1007/s12311-026-02038-7

**Published:** 2026-06-23

**Authors:** Audrey Iskandar, Maresa Buchholz, Dorota Sarwinska, Stéphanie Borel, Alexandra Durr, Mariana Atencio, Claire Ewenczyk, Anna Heinzmann, Sacha Weber, Rania Hilab, Feng Xie, Brittany Humphries, Thomas Klockgether, Marcus Grobe-Einsler, Vivian Maas, Katrin Feldmann, Stéphan Rouillon, Andreas Nadke, Elin Haf Davies, Martina Minnerop, Friedrich Erdlenbruch, Almut T. Bischoff, Thomas Klopstock, Zofia Fleszar, Sylvia Boesch, Elisabetta Indelicato, Jörg B. Schulz, Kathrin Reetz, Bernhard Michalowsky

**Affiliations:** 1 German Center for Neurodegenerative Diseases, Site Rostock/Greifswald, Patient-reported Outcomes & Health Economics Research, Greifswald, Germany; 2 Sorbonne Université, Paris Brain Institute (ICM - Institut du Cerveau), INSERM, CNRS, Assistance Publique-Hôpitaux de Paris (AP-HP), Paris, France; 3 Department of Health Research Methods, Evidence and Impact, McMaster University, Hamilton, ON Canada; 4 German Center for Neurodegenerative Diseases, Bonn, Germany; 5 Center for Neurology, Department of Parkinson Disease, Sleep and Movement Disorders, University Hospital Bonn, Bonn, Germany; 6 Department of Neurology, Center for Movement Disorders and Neuromodulation, Medical Faculty, Heinrich-Heine University, University Hospital Düsseldorf, Düsseldorf, Germany; 7 Association Française de l’Ataxie de Friedreich, Saint Avé, France; 8 Deutsche Heredo-Ataxie-Gesellschaft, Stuttgart, Germany; 9 Aparito, a wholly owned subisidiary of Eli Lilly and Company, Ty Dewi Sant, Rhosddu Road, Wrexham, Wales LL11 1NF UK; 10 Institute of Clinical Neuroscience and Medical Psychology, Medical Faculty, Heinrich-Heine-University, University Hospital Düsseldorf, Düsseldorf, Germany; 11 Research Centre Jülich, Institute of Neuroscience and Medicine (INM-1), Jülich, Germany; 12 Department of Neurology and Center for Translational Neuro- and Behavioral Sciences (C-TNBS), University Hospital Essen, University of Duisburg-Essen, Essen, Germany; 13 Department of Neurology, Ludwig-Maximilians-Universität (LMU), Friedrich-Baur-Institute, Munich, Germany; 14 German Center for Neurodegenerative Diseases (DZNE), Munich, Germany; 15 Munich Cluster for Systems Neurology (SyNergy), Munich, Germany; 16 Hertie Institute for Clinical Brain Research, University of Tuebingen, Tuebingen, Germany; 17 Department of Neurology, Center for Rare Movement Disorders Innsbruck, Medical University Innsbruck, Innsbruck, Austria; 18 Department of Neurology, RWTH Aachen University, Aachen, Germany

**Keywords:** Ataxia, Movement disorders, Adolescence well being, Health status, Life course perspective

## Abstract

Friedreich’s ataxia (FA) is a rare, autosomal recessive neurodegenerative disease that primarily affects children and adolescents in their transition to adulthood, which is associated with several adverse life events caused by the disease progression. This article aims to describe life events associated with FA and their impact on patient-reported outcomes in pediatric and adult onsets. A newly adapted Life Events Questionnaire was applied within the PROFA study. The questionnaire captured various disease-, relationship-, and work-related life events. The outcomes were stratified by pediatric and adult-onset groups at subcategory and item levels. Non-parametric tests, along with multivariate regressions with adjustments were employed. A total of 73 patients (44 pediatric-/29 adult-onset) completed the Life Events Questionnaire. Both pediatric and adult onset groups reported a similar number of life events (M_events_ = 5.3), with disease-related events being the most prevalent. Item-level analyses revealed that adult-onset had a higher likelihood in identifying life events, including positive life events, compared to pediatric-onset group. Regression analyses revealedassociations between increased disability and increased adverse life events particularly relationship-related adverse life events(IRR_assist_ = 2.044, *p* < 0.05; IRR_non−ambulant_ = 2.589, *p* < 0.01), as well as moderating effect of age at onset specifically for non-ambulant patients (IRR = 0.061, *p* < 0.01). The pediatric onset group were more prone to relationship-related adverse life events, especially in non-ambulant stage. In contrast, with adult-onset may benefit from established social identity in which their partners play a supportive role. Psychosocial support should be offered preemptively at the start of diagnosis for example concurrently with genetic counseling. The findings suggests that disease management strategies should be tailored to different age groups.

**Supplementary Information:**

The online version contains supplementary material available at 10.1007/s12311-026-02038-7.

## Introduction

Friedreich’s Ataxia (FA) is a rare, autosomal recessive neurodegenerative disease with a debilitating life-shortening effect that affects 1 in 40,000 people worldwide [1]. The disease is caused by a GAA-repeat expansion in the *FXN* (frataxine) gene located on chromosome 9 [2]. The length of the GAA1 allele is an important determinant of the clinical severity of the disease, and is inversely correlated with the age of symptom onset [2, 3]. The resulting energy deficiency and oxidative stress primarily affect the cerebellum, spinal cord, and peripheral nerves, leading to difficulties with mobility, coordination, speech, and swallowing. Individuals with FA may also experience heart and musculoskeletal problems (e.g. scoliosis, pes cavus) [4] as well as complications such as vision problems and, less commonly, hearing loss [2].

FA typically manifests in children and adolescents aged 8 to 14 years [5]. The pediatric onset (< 18 years) can profoundly interfere with growth, maturation, and psychosocial development, beyond the typical challenges of puberty. An earlier onset is also associated with faster disease progression and a higher likelihood of comorbidities [4, 6, 7], including affective disorders [8], which exacerbate the overall disease burden and may interfere with coping strategies during adolescence, and, thus, can further impair quality of life and complicate psychosocial development.

In social sciences, such challenges faced by patients in their daily lives are defined as *life events*, described as singular occurrences that mark a change in status, thereby requiring (re)adjustment and a change in an individual’s pattern of living [9]. Research by White et al. [10] on FA-related transitional life events highlighted the importance of examining both disease-related and developmental factors. Life events, along with psychosocial factors, contribute to the construct of illness perception, which refers to an individual’s beliefs about their illness and its impact on them [11]. Notably, illness perception has been identified as a modifiable factor influencing self-management in other neurological diseases with motor symptoms, such as multiple sclerosis [12].

While age stratification is a key predictor of disease progression, the emphasis on symptom-related outcomes overlooks the psychosocial challenges patients face during transitional phases. Moreover, symptom progression is often categorized into “ambulant” and “non-ambulant” (e.g [5, 13, 14]). to operationalize disease stages. However, this framework primarily addresses physical decline and overlooks the equally critical psychosocial transitions that are crucial for patients’ everyday lives. Moreover, according to Settersten et al. [15], different age groups are also associated with various roles and expectations. Therefore, age may also influence how symptoms are managed. While children may struggle with communication and dependence on caregivers, adults may deal with role disruption and chronic stress [16–18].

Given the divergent life priorities across the lifespan, disease management presents a significant challenge in adolescents and young adults [19, 20]. They are concurrently navigating key developmental milestones [21]—such as autonomy, identity formation, education, and social relationships—while also confronting the burden of progressive disability. These overlapping demands can amplify the risk of psychiatric comorbidities [22, 23] and highlight the need to consider the developmental stage when examining the impact of FA on patients’ everyday lives. The differing priorities of different age groups shape not only behaviour but also influence how individuals experience, interpret, and manage psychological or physical symptoms at various stages of life [24]. Consequently, the challenges of symptom management are age-contingent, and we, therefore, hypothesize that pediatric-onset patients will experience distinct transitional events compared to adult-onset patients, underscoring the need for targeted interventions that account for these age-related differences. In short, the central aim of this analysis was to identify the most frequently reported adverse life events in FA and demonstrate how its salience changes with increasing disease severity in patients with pediatric- and adult-onset disease.

## Methods

### Study Design and Participants

The Patient-Reported, health economic and psychosocial Outcomes in patients with Friedreich’s Ataxia (PROFA) study is an on-going observational study conducted at six European study centers in Austria, Germany, and France. The in-person data assessment in study centers was combined with an app-based, remote, patient-reported self-assessment at home [25].

Patients with a genetically confirmed diagnoses of FA, aged older than 11, with an ataxia severity of ≤ 30 on the Staging of Ataxia Rating (SARA), with access to a digital device, and the ability to use it were eligible to participate in the PROFA study. All patients or their legal guardian were informed about the study and provided written informed consent at the time of enrollment. The study was approved by the local ethics committees of each participating center, registered in the ClinicalTrials.gov Registry (NCT05943002) on 12th July 2023, and was conducted in accordance with the Declaration of Helsinki [26]. The study protocol is available elsewhere [25].

### Data Assessments and Procedures

The initial baseline assessment was conducted at each study center. It included basic sociodemographic information and clinical outcomes, along with some patient-reported measures. Participants were then instructed to download a mobile research app (Atom5™ by Aparito) on their own device (phone or tablet) specifically designed for the PROFA study. Participants were subsequently introduced to the app and invited to complete a series of measurements over the next six months on a two-monthly basis, including the newly adapted Life Events Questionnaire. This analysis utilizes the first measurement of each instrument.

### Life Events Questionnaire

An 18-item Life Events Questionnaire was adapted from the publication by White et al. (2010) [10], which listed the most frequently identified transitional life events in Friedreich’s Ataxia patients, and was implemented in the app. White et al. conducted a thematic analysis based on semi-structured interviews with 20 participants. Each interview lasted between one and two hours, allowing for a deep exploration of the lived experience. From these narratives, the researchers synthesized specific topics which were then organized into broader categorical themes representing transitional life events in Friedreich Ataxia. The questionnaire used PROFA was derived from the life event categories and topics identified by White et al., operationalizing their qualitative findings into structured items. The questionnaire comprised of six items related to disease-related events, eight items related to relationship-related events, and four items related to life’s school or work-related events. Each question can be answered with ‘no’, ‘yes’, or ‘not applicable’. In this context, disease- and relationship-related items reflect how different age groups perceive and experience the illness, as well as how the illness impacts their relationships. Life’s school or work-related events illustrate how the illness impacts aspects of professional development and its implications for future financial independence.

**Fig. 1:**
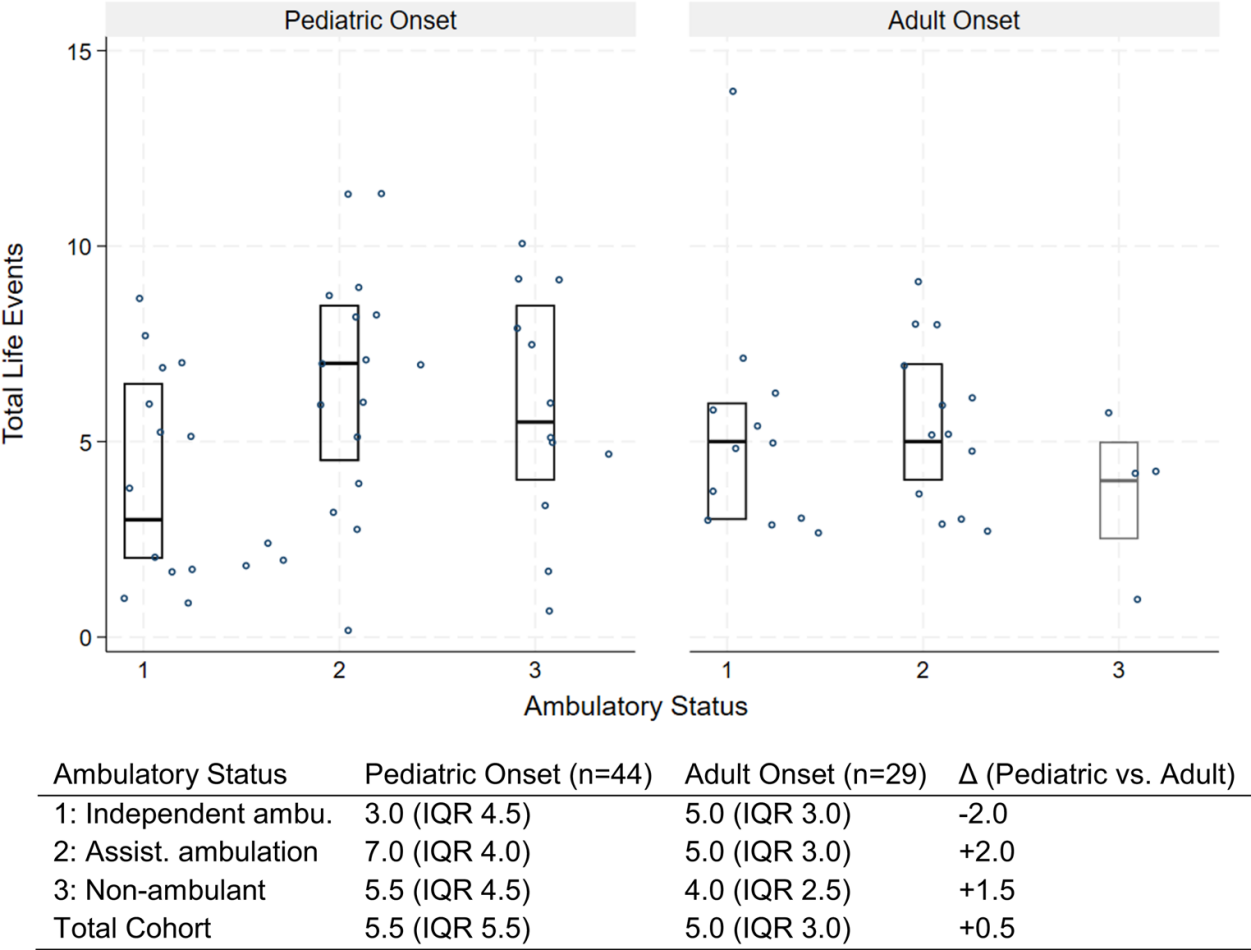
The struggle of transition as depicted by total adverse life events

**Fig. 2:**
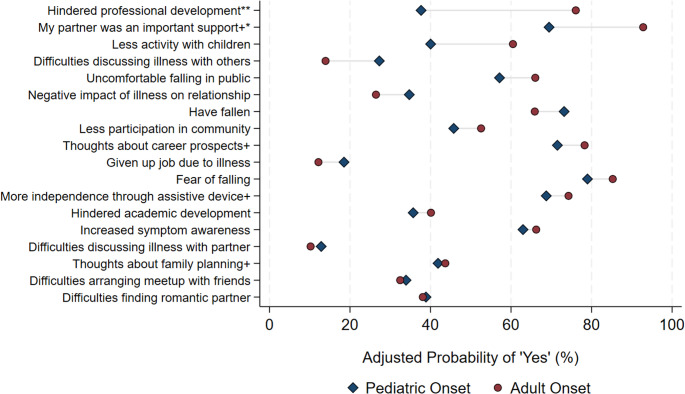
Predicted probability of life events across onset groups during the last two months. * p < 0.05, ** p < 0.01, *** p < 0.001 for difference between onset groups. Note. Items marked with + are positive formulated items (non-adverse life event). The average disability stage score for both onsets are 4.3 (SD 1.4)/ 5(IQR 3-6) and 4.2 (SD 1.2)/4 (IQR 3-5) for pediatric and adult-onset, respectively. These scores are within the level 2 of summarized ambulation status “assisted ambulation” (see Supplementary Table 1). Breakdown of predicted probabilities in Supplementary Table 4

The complete overview of the assessed life events can be found in the Supplementary materials. For group-level analyses (Fig. 1) involving the aggregation of “yes” and “no” responses, items were standardized to ensure uniform construct directionality. Specifically, positively worded items were reverse-coded to align with negatively valenced items, thereby achieving a consistent (negative) construct formulation and enabling valid summation and comparison across items. In contrast, for item-level analyses (Fig. 2), individual items were retained in their original wording and evaluated. Thus group-level analyses explicitly analyzed adverse life events while item-level analyses retained the original valence of each item.

### Life Events Across the Onset Group and Disability Stages

A composite variable of Functional Disability Staging (FDS) [27, 28] and onset group was created to capture the interaction between these two dimensions more effectively, thereby providing a nuanced view of disease progression from different age-related starting points [29]. Age at onset was categorized into pediatric-onset (PO) and adult-onset (AO), operationalized as onset before or after 18 years, respectively. The FDS were condensed from seven ordinal levels, ranging from no disability to total disability. We categorized these stages into independent ambulation (stages 1–3), assisted ambulation (stages 4 and 5), and non-ambulant living patients (stages 6 and 7), as demonstrated in the Supplementary Table 1. Due to its weight of classification on ambulatory status, the FDS will be interchangeably referred to as “ambulation” or “ambulatory status” throughout the paper.

### Associated Factors

To investigate the association between life event domains with subjectively perceived health and wel-lbeing, as well as the association between the frequency of life events and increasing disability staging as the primary marker of disease progression in FA, the following outcomes were also included in the analyses:

EQ-VAS (Visual Analogue Scale) of the EQ-5D-5 L, ranging from 0 (the worst imaginable health state) to 100 (the best imaginable health state) reporting on the participant’s current health state [30]; and the WEMWBS (Warwick-Edinburgh Mental Wellbeing Scale), a validated 14-item scale consisting of positively worded statements to monitor mental wellbeing with each item ranging from one (None of the time) to five (All of the time) with a sum score ranging from 14 to 70 [31, 32].

### Statistical Analysis

The analyses were carried out cross-sectionally, including only baseline measurements. The study population and the prevalence of life events across disability stages or onset groups were presented descriptively, along with statistical comparisons between subgroups (Mann-Whitney-U, Kruskal-Wallis, Pearson’s Chi-squared test). Visual aids such as dumbbell plot, box and bar chart were also generated to highlight the most important differences. The dumbbell plot was constructed based off of the estimated marginal probability of each “yes” answer for each item in the life events questionnaire between the onset groups. Results represent the adjusted probabilities calculated as marginal effects at mean (MEM), holding ambulatory status constant at its sample mean to isolate the effect of the onset group. Standard errors were estimated using robust variance estimators and accounted for the unconditional distribution of covariates to ensure reliability in a small sample. Additionally, a box plot shows the overall differences in the sum of the adverse life events while a bar chart was constructed to illustrate the differences between smaller subgroups in pediatric onset in order to factor in puberty.

A Poisson regression model was subsequently used to investigate factors affecting the number of adverse life events, adjusting for disability stages, age at onset, sex, years of education, and overall wellbeing. To account for potential mild overdispersion and heteroscedasticity, we utilized robust standard errors (vce robust). Results are reported as Incidence Rate Ratios (IRRs), calculated by exponentiating the raw regression coefficients. A p-value of < 0.05 was considered statistically significant. All analyses were conducted using Stata 18.0.

## Results

### Sample Description

The analysis included 73 patients out of 114 patients ranging from 13 to 67 years old (mean age 35.8 ± 14.4 (median age 34; IQR_p25−p75_ 24–44); female 50.7%; mean SARA 16.9 ± 6.1; mean GAA1 repeat expansion 562.2 ± 413.9) who completed the Life Events Questionnaire by 01st December 2025. In this sample, there was a higher number of individuals with pediatric onset of FA (*n* = 44, 60.3%) compared to those with adult onset (*n* = 29, 39.7%). The pediatric-onset group reported having a significantly younger age (28.2 vs. 47.2, *p* < 0.001) and an earlier age of FA onset (12.3 vs. 30.4, *p* < 0.001) with longer GAA1 repeat length (686.3 vs. 392, *p* < 0.001), as well as having lower WEMWBS (49.2 vs. 52.8; *p* = 0.026) compared to the adult-onset at baseline. Pediatric onset patients were mainly cared for by their parents (59.1%), while adult onset patients were cared for by their partners (60.7%; *p* = 0.002). There were no further significant differences in sociodemographic and clinical factors between both groups. The sample characteristics are presented in Table 1.

**Table 1:** Sample characteristics divided by pediatric vs. adult onset

	Onset < 18 y M (SD) / *n* (%)	Onset ≥ 18 y M (SD) / *n* (%)	Total M (SD) / *n* (%)	*p*-value
N	44 (60.3%)	29 (39.7%)	73 (100.0%)	
Sociodemographic				
Age	28.2 (10.7)	47.2 (11.6)	35.8 (14.4)	**< 0.001**
Sex*				
Male	19 (43.2%)	17 (58.6%)	36 (49.3%)	0.197
Female	25 (56.8%)	12 (41.4%)	37 (50.7%)	
Employment*				
Unemployed	26 (59.1%)	9 (31.0%)	35 (47.9%)	**0.019**
Employed	18 (40.9%)	20 (69.0%)	38 (52.1%)	
Education (years)	13.4 (3.4)	15.3 (2.9)	14.1 (3.3)	**0.004**
Children (number)	1.3 (0.7)	2.1 (0.9)	1.8 (0.9)	0.052
Clinical Outcomes				
Age at Onset	12.3 (3.7)	30.4 (11.1)	19.5 (11.7)	**< 0.001**
Disease duration	15.8 (10.5)	16.8 (8.3)	16.2 (9.6)	0.382
SARA	17.5 (6.7)	16.1 (5.2)	16.9 (6.1)	0.341
INAS	4.3 (1.9)	4.6 (1.7)	4.4 (1.8)	0.312
*FXN* GAA1	686.3 (444.9)	392.0 (298.6)	562.2 (413.9)	**< 0.001**
Total life events**	5.4 (3.0)	5.2 (2.5)	5.3 (2.8)	0.608
Disease-related **	3.0 (1.7)	3.1 (1.2)	3.0 (1.5)	0.727
Relationship-related **	1.6 (1.5)	1.3 (1.6)	1.5 (1.5)	0.197
Life’s work-related **	0.8 (0.8)	0.8 (0.8)	0.8 (0.8)	0.830
FARS-ADL	12.7 (5.5)	12.6 (3.9)	12.7 (4.9)	0.963
EQ-VAS	60.7 (21.9)	56.2 (20.7)	58.8 (21.4)	0.448
WEMWBS	49.2 (8.9)	52.8 (9.4)	50.6 (9.2)	**0.026**
Relationship with CG***				
None	4 (9.1%)	1 (3.6%)	5 (6.9%)	**0.002**
Parents	26 (59.1%)	4 (14.3%)	30 (41.7%)	
Siblings	0 (0.0%)	1 (3.6%)	1 (1.4%)	
Partner	11 (25.0%)	17 (60.7%)	28 (38.9%)	
Friends	1 (2.3%)	3 (10.7%)	4 (5.6%)	
Others	2 (4.5%)	2 (7.1%)	4 (5.6%)	

* Count variables tested with Pearson’s Chi-squared test

** Measured every two months

CGCaregiver,INASInventory of Non-Ataxia Signs. The average values showed are mean and standard deviation. The tests used are Kruskal-Wallisand Pearson's chi squared test

### Prevalence of Adverse Life Events Across Disability Stages and Onset Groups

In both onset groups, disease-related life events were the most frequently reported life events, followed by the relationship-related life events (see Table 2). In general, there were more adverse life event in average during the assisted ambulation stage (mean (M) = 6.1 life events, SD = 2.6) compared to the independent ambulation (M = 4.6, SD = 2.9) and non-ambulant stages (M = 5.3, SD = 2.8).

**Table 2:** Patient-reported outcomes across disability staging between onset groups

					Life Events				Patient-reported Outcomes		
		**N**			Total life events	Disease events	Relationship events	School/ work events	ADL	EQ-VAS	WEMWBS
PediatricOnset	Indep. ambul.			16 (36.4%)	4.1 (2.7)	2.7 (1.7)	0.8 (0.8)	0.6 (0.7)	8.0 (4.3)	77.7 (14.2)	51.6 (7.6)
	Assist. ambul.			16 (36.4%)	6.5 (3.0)	3.6 (1.7)	1.9 (1.2)	0.9 (1.1)	13.9 (3.6)	50.3 (24.7)	47.1 (9.6)
	Non-ambulant			12 (27.3%)	5.8 (2.9)	2.5 (1.3)	2.3 (2.0)	1.0 (0.6)	17.5 (3.8)	58.2 (14.8)	48.7 (9.3)
	*Total*			44 (100.0%)	5.4 (3.0)	3.0 (1.7)	1.6 (1.5)	0.8 (0.8)	12.7 (5.5)	60.7 (21.9)	49.2 (8.9)
	*p-value*				0.059	0.162	**0.016**	0.290	**< 0.001**	**0.006**	0.297
AdultOnset	Indep. ambul.		12 (41.4%)		5.3 (3.1)	3.2 (1.4)	1.5 (2.0)	0.6 (0.9)	10.5 (3.6)	59.2 (20.0)	55.3 (9.6)
	Assist. ambul.		13 (44.8%)		5.5 (2.0)	3.2 (0.9)	1.5 (1.5)	0.8 (0.7)	13.3 (3.2)	51.7 (24.3)	50.6 (10.3)
	Non-ambulant		4 (13.8%)		3.8 (2.1)	2.5 (1.7)	0.2 (0.5)	1.0 (0.8)	16.2 (3.8)	62.2 (2.1)	52.2 (4.3)
	*Total*		29 (100.0%)		5.2 (2.5)	3.1 (1.2)	1.3 (1.6)	0.8 (0.8)	12.6 (3.9)	56.2 (20.7)	52.8 (9.4)
	*p-value*				0.428	0.845	0.237	0.407	**0.035**	0.652	0.169
Total	Indep. ambul.		28 (38.4%)		4.6 (2.9)	2.9 (1.6)	1.1 (1.4)	0.6 (0.8)	9.0 (4.1)	68.0 (19.5)	53.2 (8.5)
	Assist. ambul.		29 (39.7%)		6.1 (2.6)	3.4 (1.4)	1.7 (1.3)	0.9 (0.9)	13.6 (3.4)	51.0 (24.0)	48.7 (9.9)
	Non-ambulant		16 (21.9%)		5.3 (2.8)	2.5 (1.4)	1.8 (2.0)	1.0 (0.6)	17.2 (3.7)	59.2 (12.8)	49.6 (8.4)
	*Total*		73 (100.0%)		5.3 (2.8)	3.0 (1.5)	1.5 (1.5)	0.8 (0.8)	12.7 (4.9)	58.8 (21.4)	50.6 (9.2)
	*p-value*				0.070	0.109	0.120	0.116	< 0.001	**0.014**	0.107

Group average shown is mean and SD; the significant test used was Kruskal Wallis

The pediatric (M = 5.4, SD = 3.0) and adult (M = 5.2, SD = 2.5) onset groups had similar average total of adverse life events. However, while there was no clear pattern of association between the total number of adverse life events in the adult onset group (ambulant: M = 5.3, SD = 3.1 to non-ambulant: M = 3.8, SD = 2.1; *p* = 0.428) a trend of increase was found in the pediatric onset group (ambulant: M = 4.1, SD = 2.7 to non-ambulant: M = 5.8, SD = 2.9; *p* = 0.059). This increase appears to be driven by the growth in relationship-related adverse life events across disability stages, from ambulant (M = 0.8, SD = 0.8) to non-ambulant (M = 2.3, SD = 2.0; *p* = 0.016). Notably, although not significant, the direction for the adult onset was the opposite.

Within the sample, the assisted ambulation stage was characterized by a slightly elevated number of adverse life events compared to the groups with less severe and more severe disease stages (see Fig. 1). Median comparison across ambulation stages reveal that pediatric-onset patients started with fewer adverse life events but identified more events in later stages compared to those with adult onset. Figure 2 illustrates the predicted probabilities of identifying adverse life events between the adult and pediatric onset groups. This approach estimates the probability for each group by holding disability constant at the sample average (moderate). While the adult onset group generally demonstrated a higher overall probability (> 50%) of identifying life events, they identified a broader spectrum of life events with higher probabilities, including all recorded positive life events. In contrast, despite a lower overall predicted probability, the pediatric-onset group peaked in select relationship-focused reporting, notably exceeding the rates seen in the adult-onset group.

The most pronounced between-group differences were found in life-stage specific items: the adult onset group more frequently cited hindered professional development, less activity with children, and having their partner as an important support compared to the pediatric onset group. On the other hand, the pediatric onset group showed, except for one item about having fallen down in the last two months, a higher identification with items regarding difficulties in discussing illness with others and the negative impact of FA on their relationships.

**Fig. 3:**
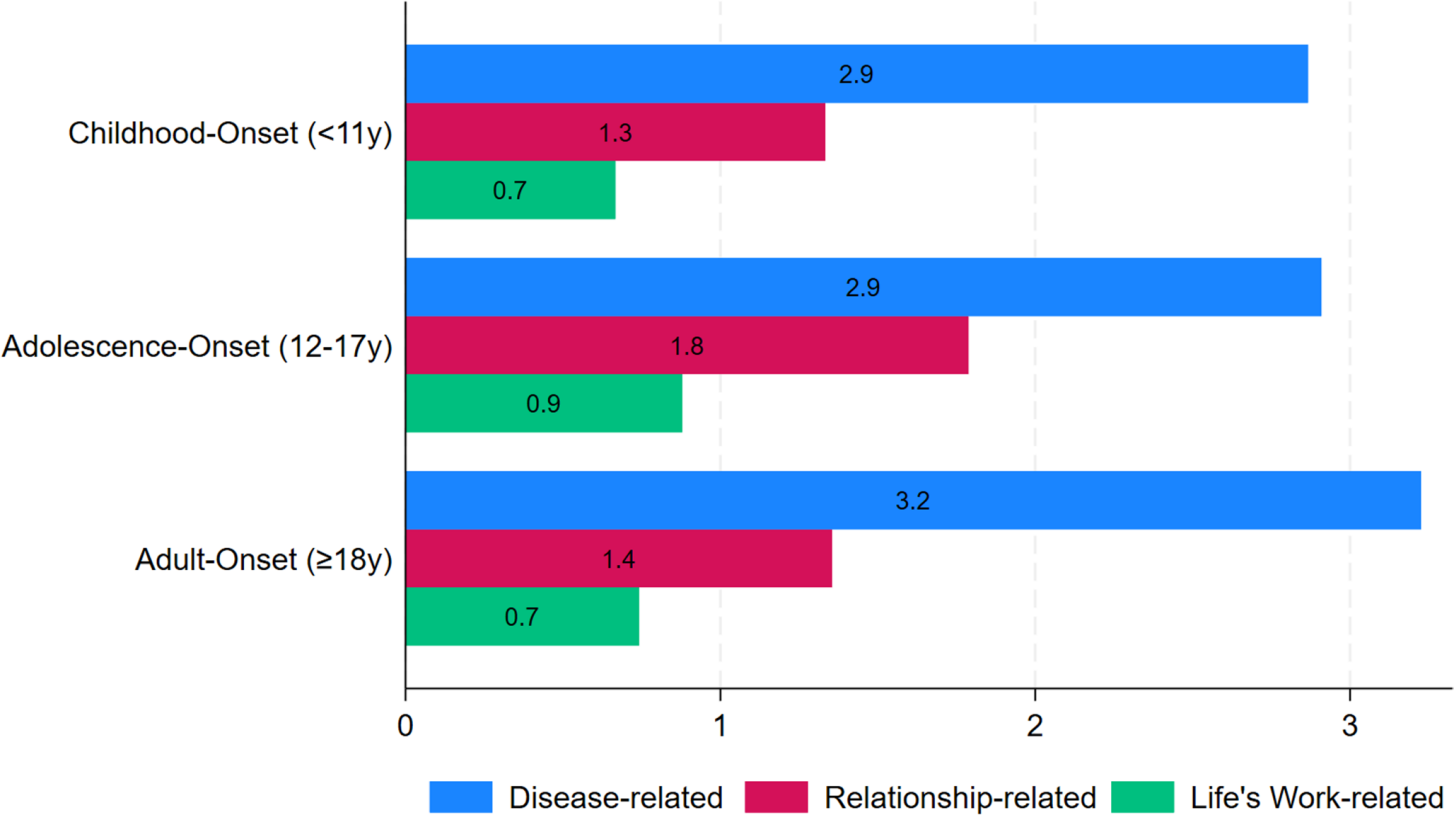
Bar chart of average life events frequency across developmental stages. The figure depicts the total average number of life events between different developmental stages. The pediatric onset was further categorized into "childhood" and "adolescence" onset

A more detailed overview of pediatric-onset cases, differentiating between onset at childhood (< 12 y) and adolescence (12–18 y), revealed additional trends. The highest average number of identified adverse life events identified in the adolescence subgroup, with relationship-related events being particularly prominent. (see Fig. 3).

### Factors associated with adverse life events

The results of the regression analyses in Table 3 demonstrated that ambulation status only marginally affected the reporting rate of the total life events in the assisted ambulation stage, increasing the reporting rate by about 47.5% (IRR = 1.475, *p* < 0.05). Specifically for relationship-related life events, the reporting rate is increased by 104.4% and 158.9% in assisted ambulation and non-ambulant stages, respectively. Additionally, the marginal interaction between ambulation stage and onset for total life events (IRR = 0.496, *p* < 0.05) indicates that the impact of losing ambulation on adverse life events was higher in pediatric onset compared to the adult onset group. Specifically, the relative rate of events for non-ambulant patients was 50.4% lower in the adult-onset group compared to the pediatric-onset group. This effect is most pronounced for relationship-related events (IRR = 0.061, *p* < 0.01) where the non-ambulant adult onset patients had 93.9% reduced rate of relationship-related adverse events compared to the non-ambulant pediatric onset.

**Table 3:** Poisson regressions of factors influencing the number of life events

	Total *N* = 73	Disease-related *N* = 73	Relationship-related *N* = 73	Life’s workrelated *N* = 73
Ambulation				
Assist. ambul.	1.475*	1.267	2.044*	1.653
Non-ambulant	1.361	0.893	2.589**	1.769
Adult onset	1.394	1.268	2.035	1.044
Ambulation* Onset				
Assist. ambul.*AO	0.647	0.735	0.408	0.87
Non-ambulant*AO	0.496*	0.833	0.061**	0.964
WEMWBS	0.983**	0.987*	0.969**	0.998
Intercept	2.639***	1.647***	2.008*	0.725
Chi-squared (χ²)	23.03	10.21	30.17	3.74
Model *p*	0.001	0.116	0.000	0.712
Pseudo R^2^	0.061	0.028	0.118	0.019

* p < 0.05, ** p < 0.01, *** p < 0.001

*AO* Adult Onset; Coefficients are shown in IRR. All models were estimated using Poisson regression with Huber-White robust standard errors to account for potential overdispersion. Marginal effect estimate can be observed in Supplementary Table 3

Furthermore, rate of adverse life events significantly lower with higher well-being scores (IRR = 0.983, *p* < 0.01); each unit increase in well-being was associated with a 1.7% reduction in the expected number of identified adverse life events. This effect of WEMWBS was replicated in the disease-related (IRR = 0.987, *p* < 0.05) and relationship-related (IRR = 0.969, *p* < 0.01) life events subcategories with 1.3% and 3.1% reduction, respectively.

## Discussion

This analysis quantified for the first time life events and their impact on patient-reported outcomes across the disease progression for different age of onset groups in FA. The majority of our sample comprised of pediatric onset cases, with 60.3% diagnosed before the age of 18. Our findings highlight distinct patterns of life events in FA depending on age at onset. While there was no association among adult-onset patients with the number life events across disability stages, those with pediatric-onset showed a trend in increase, particularly in relationship-related events. Importantly, these relationship-related events were most frequent among individuals at more advanced disability stages, coupled with lower levels of mental wellbeing. Across disability stages, our findings confirmed the salience of relationship-related life events and demonstrate that the reporting rate of pediatric-onset patients who became non-ambulant exceeded those with adult-onset. This suggests that early-onset disease may intensify the psychosocial challenges of forming and maintaining intimate and social relationships at developmentally critical life stages [19, 22].

Although the median number of identified life events peaked during the assistive ambulation stage for the pediatric-onset group, Poisson regression analyses identified the non-ambulant stage as the point of divergence between onset groups (*p* < 0.05). For the pediatric onset group, this indicates that while the assistive stage is marked by high individual variability the transition to the non-ambulant stage reveals a sharp divergence, particularly in relationship-related life events. In contrast, the adult-onset group displays a more attenuated reporting pattern. Together, this suggests that the loss of ambulation may carry a more profound psychosocial impact for those with pediatric-onset disease.

Our results align with and extend previous qualitative studies that highlight the profound psychosocial challenges associated with FA. White et al. (2010) [10] reported that transitions in mobility, relationships, and occupational roles were perceived as critical life events by patients with FA.

The findings highlight the relatively greater weight of relationship-centered experiences in pediatric-onset cases. Across life-event items, pediatric-onset individuals reported relationship-specific events more frequently than adult-onset individuals, even if their overall probability of reporting individual events is modest. This pattern may reflect social or developmental factors: adult-onset individuals may experience a more distinct disruption to their established life circumstances, resulting in higher general event reporting, whereas pediatric-onset individuals emphasize relationship-centered experiences. Mature social networks in adult-onset patients act as a vital buffer against stress [33, 34], potentially mitigating relationship issues even as physical disability worsens. In contrast, pediatric-onset patients often lack this established support. The difficulty of communicating rising needs alongside an unformed social identity can negatively impact their relationships which is reflected in the item-level analyses. This onset-dependent divergence has been underexplored in previous studies, highlighting the importance of considering disease onset in psychosocial research and clinical care.

Further, it may be crucial to address the potential stigma surrounding assistive devices; while our sample reflect a relatively high acceptance of assistive device, current research suggests that some individuals with FA postpone their use, choosing to rely on others or avoid public spaces entirely rather than utilize a cane [28]. Ejaz et al. [35] noted that mobility devices correlate with a decline in quality of life domains for children, potentially indicating that for younger patients, the device is viewed as a marker of disability rather than a facilitator of autonomy [36].

The association between relationship-related events and lower well-being aligns with prior research, which suggests that the simultaneous strain of progressing disability and navigating developmental milestones heightens the risk of psychiatric comorbidities [22, 23]. The FA guideline on mental health emphasized that disease-related transitions often exacerbate grief and loss and compromise identity and psychological resilience [37]. Similarly, qualitative work has described psychosocial challenges as pervasive in everyday life with FA, highlighting loss, adaptation, and shifting sources of strength [38]. Our findings contribute to this body of evidence by providing quantitative evidence to support that adverse relationship events are not only frequent but also significantly associated with well being.

Combined, these findings indicate that patients with pediatric-onset FA face distinct psychosocial challenges, highlighting the need for tailored approaches in disease management. Thus, interpersonal challenges are particularly detrimental to pediatric onset patients, as the developmental process is deeply intertwined with socialization processes [39]. Taken together, our results further corroborate existing literature such as Settersten’s age-structuring theory [15] and highlight varying illness perceptions and associated role disruptions in different age groups with varying onset ages at various developmental stages [21], for which different management strategies are needed [16, 17].

### Limitations

This study is the first to quantify psychosocial consequences, offering new insights into disease management across age groups. Future work should not only address content and construct validity, but also examine the implications of non-applicable (NA) ratings in the Life Events Questionnaire, and incorporate the differential psychosocial influence of FA in different age groups. Due to the frequency of identified relationship-related events, patients’ interaction with their caregivers should be investigated more closely, especially for patients with a pediatric onset. Longitudinal analyses are essential for capturing how life events unfold over time and for disentangling the causal relationships between adverse life events and other psychosocial aspects. Furthermore, the lack of a control group from the general population is a limitation of this study. Future research should incorporate comparative data from national cohorts—such as those in Germany, France, or Austria—to account for regional and cultural variations in the prevalence of life events.

## Conclusion

This article examines how the age at onset shapes the lived experience of FA by investigating reported life events across the transition from independent walking to non-ambulant stages. Aside from the negative effect on well-being, our findings highlight distinct onset-dependent trajectories: there is a trend in which adult-onset patients reported fewer life events in average as disability progressed, whereas pediatric-onset patients experienced a significant increase, particularly in relationship-related events. Moreover, despite the lower predicted probability of identifying a life event compared to the adult onset, the pediatric onset have 93.9% higher reporting rate for relationship-related life events in non-ambulant stage compared to the adult onset suggesting that the developmental timing of disease onset profoundly shapes the psychosocial burden of FA. Additionally, the items in which pediatric onset’s reporting probability exceeds the adult onset are related to disease communication and the negative impact of FA in interpersonal relationships. Combined together, these results implies that pediatric FA patients are especially vulnerable due to the combined burden of developmental changes, which include interpersonal challenges, together with FA symptoms. Therefore, psychosocial support must be preemptive rather than reactive. Because the subjective impact intensifies significantly in later stages, intervention should be offered at the point of diagnosis or genetic counseling. While adult-onset patients may benefit from practical support—such as professional development and domestic assistance (e.g., childcare)—the pediatric group requires earlier support focused on identity formation and the internalization of the chronic illness. Specifically, counseling initiated at the point of genetic testing should address the de-stigmatization of assistive devices to mitigate the psychological burden as the disease progresses.

The results underscore the importance of incorporating onset-specific considerations into clinical care and support strategies. Future studies should aim to gain a deeper understanding of the psychosocial burden of FA across the lifespan. By considering the unique pathways of patients in different life stages, researchers can tailor interventions to address differing illness perceptions across various age at onset. Comparative studies across different hereditary ataxias, such as spinocerebellar ataxias, could clarify whether the onset-dependent patterns we observed are specific to FA or represent broader features of progressive ataxic disorders. Moreover, qualitative approaches should complement quantitative measures to deepen insight into patients’ lived experiences, particularly regarding interpersonal challenges and the use of assistive devices. Finally, future intervention studies should include targeted psychosocial support, counselling, or stigma-reduction strategies can mitigate the adverse impact of relationship-related life events and improve mental wellbeing in FA, especially for those with pediatric onset.

## Electronic Supplementary Material

Below is the link to the electronic supplementary material.

Supplementary Material 1

## Data Availability

The data that support the findings of this study are available on request from the corresponding author. The data are not publicly available due to privacy or ethical restrictions.
