# Supplementary material for: Transitional Life Events in Friedreich Ataxia: Differential Age at Onset Perspectives
Source: Cerebellum. 2026 Jun 23;25(4):96. doi: 10.1007/s12311-026-02038-7 (PMC13291036; doi:10.1007/s12311-026-02038-7)
Supplement: Supplementary file 1 — Supplementary Material 1 [file 12311_2026_2038_MOESM1_ESM.docx]

**Electronic supplementary materials**

## Supplementary Table 1. Adapted Functional Disability Staging

|  | Functional Disability Staging |  | Summarized disability stages |
| --- | --- | --- | --- |
| 1  2  3 | No functional handicap but signs at examination  Mild, able to run, walking unlimited  Moderate, unable to run, limited walking without aid | 1 | Independent ambulation/  /Ambulant/Walking |
| 4  5 | Severe, walking with one stick  Walking with two sticks | 2 | Assisted ambulation/  Walking with assistance |
| 6  7 | Unable to walk, requiring wheelchair  Confined to bed | 3 | Non-ambulant |

## Supplementary Table 2. Detailed breakdown of adverse life events across disability staging and onset groups

***In the last two months*…**

|  | **Pediatric Onset** | | | | |  | **Adult Onset** | | | | |
| --- | --- | --- | --- | --- | --- | --- | --- | --- | --- | --- | --- |
|  | Indep. Ambul. | Assist. Ambul. | Non-ambulant | Total | Test |  | Indep. Ambul. | Assist. Ambul. | Non-ambulant | Total | Test |
| N | 16 (36.4%) | 16 (36.4%) | 12 (27.3%) | 44 (100.0%) |  | N | 12 (41.4%) | 13 (44.8%) | 4 (13.8%) | 29 (100.0%) |  |
| **Disease-related** | | | | |  |  | | | | |  |
| *I have become particularly aware of my symptoms* | | | | |  | *I have become particularly aware of my symptoms* | | | | |  |
| No | 5 (29.4%) | 5 (29.4%) | 2 (15.4%) | 12 (25.5%) | 0.647 | No | 4 (30.8%) | 6 (42.9%) | 0 (0.0%) | 10 (32.3%) | 0.572 |
| Yes | 6 (35.3%) | 9 (52.9%) | 7 (53.8%) | 22 (46.8%) |  | Yes | 8 (61.5%) | 7 (50.0%) | 3 (75.0%) | 18 (58.1%) |  |
| N/A | 6 (35.3%) | 3 (17.6%) | 4 (30.8%) | 13 (27.7%) |  | N/A | 1 (7.7%) | 1 (7.1%) | 1 (25.0%) | 3 (9.7%) |  |
| *I have fallen e.g. at home or on the road* | | | |  |  | *I have fallen e.g. at home or on the road* | | | |  |  |
| No | 5 (29.4%) | 3 (17.6%) | 4 (30.8%) | 12 (25.5%) | 0.930 | No | 4 (30.8%) | 5 (35.7%) | 1 (25.0%) | 10 (32.3%) | 0.392 |
| Yes | 11 (64.7%) | 13 (76.5%) | 8 (61.5%) | 32 (68.1%) |  | Yes | 9 (69.2%) | 9 (64.3%) | 2 (50.0%) | 20 (64.5%) |  |
| N/A | 1 (5.9%) | 1 (5.9%) | 1 (7.7%) | 3 (6.4%) |  | N/A | 0 (0.0%) | 0 (0.0%) | 1 (25.0%) | 1 (3.2%) |  |
| *I was afraid of falling* | |  |  |  |  | *I was afraid of falling* | |  |  |  |  |
| No | 4 (23.5%) | 3 (17.6%) | 2 (15.4%) | 9 (19.1%) | 0.931 | No | 3 (23.1%) | 0 (0.0%) | 1 (25.0%) | 4 (12.9%) | **0.026** |
| Yes | 11 (64.7%) | 13 (76.5%) | 9 (69.2%) | 33 (70.2%) |  | Yes | 8 (61.5%) | 14 (100.0%) | 2 (50.0%) | 24 (77.4%) |  |
| N/A | 2 (11.8%) | 1 (5.9%) | 2 (15.4%) | 5 (10.6%) |  | N/A | 2 (15.4%) | 0 (0.0%) | 1 (25.0%) | 3 (9.7%) |  |
| *I felt uncomfortable falling in front of others* | | | |  |  | *I felt uncomfortable falling in front of others* | | | |  |  |
| No | 6 (35.3%) | 6 (35.3%) | 4 (30.8%) | 16 (34.0%) | 0.270 | No | 3 (23.1%) | 3 (21.4%) | 1 (25.0%) | 7 (22.6%) | 0.877 |
| Yes | 7 (41.2%) | 10 (58.8%) | 4 (30.8%) | 21 (44.7%) |  | Yes | 7 (53.8%) | 6 (42.9%) | 1 (25.0%) | 14 (45.2%) |  |
| N/A | 4 (23.5%) | 1 (5.9%) | 5 (38.5%) | 10 (21.3%) |  | N/A | 3 (23.1%) | 5 (35.7%) | 2 (50.0%) | 10 (32.3%) |  |
| *I have participated less in community activities due to my illness* | | | | | | *I have participated less in community activities due to my illness* | | | | | |
| No | 8 (47.1%) | 7 (41.2%) | 9 (69.2%) | 24 (51.1%) | 0.359 | No | 7 (53.8%) | 4 (28.6%) | 3 (75.0%) | 14 (45.2%) | 0.217 |
| Yes | 6 (35.3%) | 9 (52.9%) | 4 (30.8%) | 19 (40.4%) |  | Yes | 6 (46.2%) | 10 (71.4%) | 1 (25.0%) | 17 (54.8%) |  |
| N/A | 3 (17.6%) | 1 (5.9%) | 0 (0.0%) | 4 (8.5%) |  | N/A | 0 (0.0%) | 0 (0.0%) | 0 (0.0%) | 0 (0.0%) |  |
| *I was more independent in everyday life through the use of assistive devices (e.g. walker, wheelchair)** | | | | | | *I was more independent in everyday life through the use of assistive devices (e.g. walker, wheelchair)** | | | | | |
| No | 5 (29.4%) | 5 (29.4%) | 0 (0.0%) | 10 (21.3%) | **0.013** | No | 6 (46.2%) | 0 (0.0%) | 1 (25.0%) | 7 (22.6%) | **0.027** |
| Yes | 5 (29.4%) | 11 (64.7%) | 9 (69.2%) | 25 (53.2%) |  | Yes | 4 (30.8%) | 11 (78.6%) | 2 (50.0%) | 17 (54.8%) |  |
| N/A | 7 (41.2%) | 1 (5.9%) | 4 (30.8%) | 12 (25.5%) |  | N/A | 3 (23.1%) | 3 (21.4%) | 1 (25.0%) | 7 (22.6%) |  |
| **Relationship-related** | | | |  |  |  | | | |  |  |
| *I had difficulties meeting up with friends* | | | |  |  | *I had difficulties meeting up with friends* | | | |  |  |
| No | 10 (58.8%) | 9 (52.9%) | 8 (61.5%) | 27 (57.4%) | 0.447 | No | 9 (69.2%) | 7 (50.0%) | 3 (75.0%) | 19 (61.3%) | 0.523 |
| Yes | 3 (17.6%) | 7 (41.2%) | 4 (30.8%) | 14 (29.8%) |  | Yes | 4 (30.8%) | 4 (28.6%) | 1 (25.0%) | 9 (29.0%) |  |
| N/A | 4 (23.5%) | 1 (5.9%) | 1 (7.7%) | 6 (12.8%) |  | N/A | 0 (0.0%) | 3 (21.4%) | 0 (0.0%) | 3 (9.7%) |  |
| *I have had difficulties finding a romantic partner* | | | | |  | *I have had difficulties finding a romantic partner* | | | | |  |
| No | 8 (47.1%) | 6 (35.3%) | 5 (38.5%) | 19 (40.4%) | 0.845 | No | 3 (23.1%) | 5 (35.7%) | 0 (0.0%) | 8 (25.8%) | 0.565 |
| Yes | 3 (17.6%) | 6 (35.3%) | 3 (23.1%) | 12 (25.5%) |  | Yes | 3 (23.1%) | 2 (14.3%) | 0 (0.0%) | 5 (16.1%) |  |
| N/A | 6 (35.3%) | 5 (29.4%) | 5 (38.5%) | 16 (34.0%) |  | N/A | 7 (53.8%) | 7 (50.0%) | 4 (100.0%) | 18 (58.1%) |  |
| *I have had difficulties talking to others about my illness progression* | | | | | | *I have had difficulties talking to others about my illness progression* | | | | | |
| No | 12 (70.6%) | 9 (52.9%) | 8 (61.5%) | 29 (61.7%) | 0.233 | No | 10 (76.9%) | 11 (78.6%) | 4 (100.0%) | 25 (80.6%) | 1.000 |
| Yes | 1 (5.9%) | 6 (35.3%) | 4 (30.8%) | 11 (23.4%) |  | Yes | 2 (15.4%) | 2 (14.3%) | 0 (0.0%) | 4 (12.9%) |  |
| N/A | 4 (23.5%) | 2 (11.8%) | 1 (7.7%) | 7 (14.9%) |  | N/A | 1 (7.7%) | 1 (7.1%) | 0 (0.0%) | 2 (6.5%) |  |
| *My partner was an important support for me** | | | | |  | *My partner was an important support for me** | | | | |  |
| No | 3 (17.6%) | 2 (11.8%) | 1 (7.7%) | 6 (12.8%) | 0.511 | No | 1 (7.7%) | 1 (7.1%) | 0 (0.0%) | 2 (6.5%) | 0.617 |
| Yes | 3 (17.6%) | 7 (41.2%) | 6 (46.2%) | 16 (34.0%) |  | Yes | 10 (76.9%) | 11 (78.6%) | 2 (50.0%) | 23 (74.2%) |  |
| N/A | 11 (64.7%) | 8 (47.1%) | 6 (46.2%) | 25 (53.2%) |  | N/A | 2 (15.4%) | 2 (14.3%) | 2 (50.0%) | 6 (19.4%) |  |
| *I had difficulties talking to my partner about my illness* | | | | |  | *I had difficulties talking to my partner about my illness* | | | | |  |
| No | 5 (29.4%) | 8 (47.1%) | 6 (46.2%) | 19 (40.4%) | 0.274 | No | 8 (61.5%) | 9 (64.3%) | 2 (50.0%) | 19 (61.3%) | 0.645 |
| Yes | 0 (0.0%) | 1 (5.9%) | 2 (15.4%) | 3 (6.4%) |  | Yes | 2 (15.4%) | 0 (0.0%) | 0 (0.0%) | 2 (6.5%) |  |
| N/A | 12 (70.6%) | 8 (47.1%) | 5 (38.5%) | 25 (53.2%) |  | N/A | 3 (23.1%) | 5 (35.7%) | 2 (50.0%) | 10 (32.3%) |  |
| *My illness has had a negative impact on my relationship* | | | | | | *My illness has had a negative impact on my relationship* | | | | | |
| No | 7 (41.2%) | 6 (35.3%) | 1 (7.7%) | 14 (29.8%) | **0.001** | No | 7 (53.8%) | 8 (57.1%) | 2 (50.0%) | 17 (54.8%) | 0.972 |
| Yes | 0 (0.0%) | 2 (11.8%) | 8 (61.5%) | 10 (21.3%) |  | Yes | 2 (15.4%) | 2 (14.3%) | 0 (0.0%) | 4 (12.9%) |  |
| N/A | 10 (58.8%) | 9 (52.9%) | 4 (30.8%) | 23 (48.9%) |  | N/A | 4 (30.8%) | 4 (28.6%) | 2 (50.0%) | 10 (32.3%) |  |
| *I was less able to play or do activities with my child* | | | | |  | *I was less able to play or do activities with my child* | | | | |  |
| No | 2 (11.8%) | 3 (17.6%) | 1 (7.7%) | 6 (12.8%) | 0.252 | No | 4 (30.8%) | 3 (21.4%) | 0 (0.0%) | 7 (22.6%) | 0.411 |
| Yes | 0 (0.0%) | 3 (17.6%) | 3 (23.1%) | 6 (12.8%) |  | Yes | 3 (23.1%) | 5 (35.7%) | 0 (0.0%) | 8 (25.8%) |  |
| N/A | 15 (88.2%) | 11 (64.7%) | 9 (69.2%) | 35 (74.5%) |  | N/A | 6 (46.2%) | 6 (42.9%) | 4 (100.0%) | 16 (51.6%) |  |
| *I have given some thoughts to family planning** | | | | |  | *I have given some thoughts to family planning** | | | | |  |
| No | 5 (29.4%) | 5 (29.4%) | 5 (38.5%) | 15 (31.9%) | 0.796 | No | 4 (30.8%) | 4 (28.6%) | 0 (0.0%) | 8 (25.8%) | 0.639 |
| Yes | 3 (17.6%) | 4 (23.5%) | 4 (30.8%) | 11 (23.4%) |  | Yes | 3 (23.1%) | 3 (21.4%) | 0 (0.0%) | 6 (19.4%) |  |
| N/A | 9 (52.9%) | 8 (47.1%) | 4 (30.8%) | 21 (44.7%) |  | N/A | 6 (46.2%) | 7 (50.0%) | 4 (100.0%) | 17 (54.8%) |  |
| **Life’s work-related** | | | | | |  | | | | | |
| *My illness has hindered my development at school (e.g. socializing with classmates, performance, absence)* | | | | | | *My illness has hindered my development at school (e.g. socializing with classmates, performance, absence)* | | | | | |
| No | 8 (47.1%) | 6 (35.3%) | 4 (30.8%) | 18 (38.3%) | 0.744 | No | 2 (15.4%) | 1 (7.1%) | 0 (0.0%) | 3 (9.7%) | 0.564 |
| Yes | 3 (17.6%) | 5 (29.4%) | 2 (15.4%) | 10 (21.3%) |  | Yes | 0 (0.0%) | 1 (7.1%) | 1 (25.0%) | 2 (6.5%) |  |
| N/A | 6 (35.3%) | 6 (35.3%) | 7 (53.8%) | 19 (40.4%) |  | N/A | 11 (84.6%) | 12 (85.7%) | 3 (75.0%) | 26 (83.9%) |  |
| *My illness has hindered me in my professional development (e.g. promotion)* | | | | | | *My illness has hindered me in my professional development (e.g. promotion)* | | | | | |
| No | 8 (47.1%) | 7 (41.2%) | 4 (30.8%) | 19 (40.4%) | 0.440 | No | 4 (30.8%) | 0 (0.0%) | 1 (25.0%) | 5 (16.1%) | 0.147 |
| Yes | 3 (17.6%) | 3 (17.6%) | 6 (46.2%) | 12 (25.5%) |  | Yes | 4 (30.8%) | 9 (64.3%) | 2 (50.0%) | 15 (48.4%) |  |
| N/A | 6 (35.3%) | 7 (41.2%) | 3 (23.1%) | 16 (34.0%) |  | N/A | 5 (38.5%) | 5 (35.7%) | 1 (25.0%) | 11 (35.5%) |  |
| *I have thought about my career options** | | | |  |  | *I have thought about my career options** | | | |  |  |
| No | 4 (23.5%) | 5 (29.4%) | 2 (15.4%) | 11 (23.4%) | 0.831 | No | 2 (15.4%) | 1 (7.1%) | 1 (25.0%) | 4 (12.9%) | 0.655 |
| Yes | 9 (52.9%) | 10 (58.8%) | 9 (69.2%) | 28 (59.6%) |  | Yes | 5 (38.5%) | 8 (57.1%) | 1 (25.0%) | 14 (45.2%) |  |
| N/A | 4 (23.5%) | 2 (11.8%) | 2 (15.4%) | 8 (17.0%) |  | N/A | 6 (46.2%) | 5 (35.7%) | 2 (50.0%) | 13 (41.9%) |  |
| *I had to give up my job* | | |  |  |  | *I had to give up my job* | | |  |  |  |
| No | 10 (58.8%) | 8 (47.1%) | 7 (53.8%) | 25 (53.2%) | 0.312 | No | 6 (46.2%) | 8 (57.1%) | 2 (50.0%) | 16 (51.6%) | 0.744 |
| Yes | 0 (0.0%) | 3 (17.6%) | 3 (23.1%) | 6 (12.8%) |  | Yes | 2 (15.4%) | 0 (0.0%) | 0 (0.0%) | 2 (6.5%) |  |
| N/A | 7 (41.2%) | 6 (35.3%) | 3 (23.1%) | 16 (34.0%) |  | N/A | 5 (38.5%) | 6 (42.9%) | 2 (50.0%) | 13 (41.9%) |  |

*Note*. ***** positive life events; N/A: does not apply (to me). The statistical test used was Fischer-Exact test

## Supplementary Table 3. Estimated marginal mean of life events across disability stages by onset group

|  | Total | Disease | Relationship | Life’s work |
| --- | --- | --- | --- | --- |
|  | b/SE | b/SE | b/SE | b/SE |
| **Independent ambulation** |  |  |  |  |
| Pediatric Onset | 4.15 | 2.73 | 0.85 | 0.56 |
|  | (0.67) | (0.42) | (0.22) | (0.18) |
| Adult Onset | 5.78 | 3.46 | 1.73 | 0.59 |
|  | (0.79) | (0.34) | (0.55) | (0.25) |
| **Assisted ambulation** |  |  |  |  |
| Pediatric Onset | 6.12 | 3.46 | 1.74 | 0.93 |
|  | (0.65) | (0.38) | (0.26) | (0.26) |
| Adult Onset | 5.52 | 3.23 | 1.44 | 0.85 |
|  | (0.48) | (0.27) | (0.34) | (0.18) |
| **Non-ambulant** |  |  |  |  |
| Pediatric Onset | 5.65 | 2.44 | 2.20 | 1.00 |
|  | (0.85) | (0.39) | (0.55) | (0.17) |
| Adult Onset | 3.90 | 2.57 | 0.27 | 1.00 |
|  | (0.96) | (0.82) | (0.23) | (0.35) |

Note. **b** = The **Predicted Mean Count** of events; **SE** = The **Standard Error** of the prediction (Delta-method standard error)

## Supplementary Table 4. Adjusted Predicted Probabilities of Adverse Events by Onset Group

| Life event | Pediatric Onset  Pred. probabil. | Adult Onset  Pred. probabil. | Difference  Pred. probabil. | *p* |
| --- | --- | --- | --- | --- |
| Hindered professional development | 37.7 | 76.1 | 38.4 | **0.003** |
| My partner was an important support | 69.5 | 92.8 | 23.4 | **0.041** |
| Less activity with children | 40.0 | 60.5 | 20.5 | 0.262 |
| Difficulties discussing illness with others | 27.3 | 13.9 | 13.4 | 0.153 |
| Uncomfortable falling in public | 57.1 | 66.0 | 8.9 | 0.510 |
| Negative impact of illness on relationship | 34.7 | 26.4 | 8.3 | 0.556 |
| Have fallen | 73.2 | 65.9 | 7.3 | 0.516 |
| Less participation in community | 45.8 | 52.6 | 6.8 | 0.557 |
| Thoughts about career prospects | 71.5 | 78.3 | 6.8 | 0.597 |
| Given up job due to illness | 18.5 | 12.2 | 6.3 | 0.543 |
| Fear of falling | 79.0 | 85.3 | 6.3 | 0.513 |
| More independence through assistive device | 68.7 | 74.3 | 5.5 | 0.617 |
| Hindered academic development | 35.7 | 40.1 | 4.4 | 0.838 |
| Increased symptom awareness | 63.0 | 66.3 | 3.3 | 0.783 |
| Difficulties discussing illness with partner | 12.8 | 10.2 | 2.6 | 0.757 |
| Thoughts about family planning | 41.9 | 43.7 | 1.8 | 0.921 |
| Difficulties arranging meetup with friends | 33.9 | 32.5 | 1.4 | 0.901 |
| Difficulties finding romantic partner | 38.9 | 38.1 | 0.8 | 0.964 |

Note. Comparison of onset groups holding ambulatory status at its observed distribution
